# The Systematic Evaluation of Identifying the Infarct Related Artery Utilizing Cardiac Magnetic Resonance in Patients Presenting with ST-Elevation Myocardial Infarction

**DOI:** 10.1371/journal.pone.0169108

**Published:** 2017-01-06

**Authors:** Carine E. Hamo, Igor Klem, Sunil V. Rao, Vincent Songco, Samer Najjar, Edward G. Lakatta, Subha V. Raman, Robert A. Harrington, John F. Heitner

**Affiliations:** 1 Department of Medicine, Stony Brook University Hospital, Stony Brook New York, United States of America; 2 The Duke Clinical Research Institute, Durham, North Carolina, United States of America; 3 Division of Cardiology, New York Methodist Hospital, Brooklyn, New York, United States of America; 4 MedStar Health Research Institute, Washington, DC, United States of America; 5 Intramural Research Program, National Institute of Aging, the National Institute of Health, Baltimore, Maryland, United States of America; 6 Division of Cardiovascular Medicine, Ohio State University, Columbus, Ohio, United States of America; 7 Department of Medicine, Stanford University, Palo Alto, California, United States of America; University of Bologna, ITALY

## Abstract

**Background:**

Identification of the infarct-related artery (IRA) in patients with STEMI using coronary angiography (CA) is often based on the ECG and can be challenging in patients with severe multi-vessel disease. The current study aimed to determine how often percutaneous intervention (PCI) is performed in a coronary artery different from the artery supplying the territory of acute infarction on cardiac magnetic resonance imaging (CMR).

**Methods:**

We evaluated 113 patients from the Reduction of infarct Expansion and Ventricular remodeling with Erythropoetin After Large myocardial infarction (REVEAL) trial, who underwent CMR within 4±2 days of revascularization. Blinded reviewers interpreted CA to determine the IRA and CMR to determine the location of infarction on a 17-segment model. In patients with multiple infarcts on CMR, acuity was determined with T2-weighted imaging and/or evidence of microvascular obstruction.

**Results:**

A total of 5 (4%) patients were found to have a mismatch between the IRA identified on CMR and CA. In 4/5 cases, there were multiple infarcts noted on CMR. Thirteen patients (11.5%) had multiple infarcts in separate territories on CMR with 4 patients (3.5%) having multiple acute infarcts and 9 patients (8%) having both acute and chronic infarcts.

**Conclusions:**

In this select population of patients, the identification of the IRA by CA was incorrect in 4% of patients presenting with STEMI. Four patients with a mismatch had an acute infarction in more than one coronary artery territory on CMR. The role of CMR in patients presenting with STEMI with multi-vessel disease on CA deserves further investigation.

## Introduction

The ACC/AHA and ESC/EACTS guidelines currently give a class I recommendation to intervene on the infarct related artery (IRA) in patients presenting with ST-Elevation Myocardial Infarction (STEMI).[1, 2] In a recently published update, the recommendation for percutaneous intervention (PCI) of a non-infarct artery was changed from class III to class IIb, indicating that it may be considered in patients with STEMI and multi-vessel disease (MVD).[3] The ESC/EACTS guidelines provide a class IIa recommendation for staged revascularization on non-culprit lesions in the setting of MVD and a class IIb recommendation for immediate revascularization of significant non-culprit lesions during primary PCI.[2] However the specifics of which additional arteries to intervene upon as well as the timing of intervention remain unclear. The IRA is identified by assessing the characteristics of coronary stenosis, the flow of blood through the stenosis, the presence of a thrombus during coronary angiography (CA) as well as localization of the ST-segment elevations on ECG. The ability to identify the IRA pre-PCI in patients presenting with STEMI has not been systematically studied. Over the past decade, cardiac magnetic resonance (CMR) has emerged as the gold standard modality for detection of myocardial scar, enabling the identification of infarcts as small as 1gm, in both the acute and chronic settings.[4]

The Reduction of infarct Expansion and Ventricular remodeling with Erythropoetin After Large myocardial infarction (REVEAL) trial was a randomized placebo-controlled trial assessing the efficacy of intravenous erythropoietin on infarct size in patients with acute STEMI.[5] The trial provides a dataset in which to evaluate the accuracy of identifying the IRA during primary PCI. The aims of this descriptive study were to determine (1) how often PCI is performed in a coronary artery different from the artery supplying the territory of acute infarction identified by CMR, (2) the incidence of multiple infarcts in patients presenting with their first STEMI, and (3) the incidence of right ventricular infarcts by CMR in patients presenting with inferior STEMI.

## Materials and Methods

### Patient population

The design of REVEAL has been published previously.[5, 6] Briefly, REVEAL was a randomized, double-blind, placebo-controlled, multicenter trial examining the effects of intravenous epoetin alfa on infarct size and left ventricular remodeling in patients with STEMI. Subjects were eligible for enrollment if they presented with acute STEMI due to total occlusion (TIMI flow grade 0–1) of a major epicardial coronary artery or large branch vessel and underwent successful (<50% residual lesion or TIMI 2–3) primary or rescue angioplasty within 8 hours of onset of ischemic symptoms. Subjects with a history of left ventricular systolic dysfunction (left ventricular ejection fraction (LVEF) <50%), myocardial infarction (MI), coronary artery bypass graft (CABG), or prior PCI in the IRA were excluded due to potential confounding of infarct size measurement.[5] In the present study, a total of 113 subjects who underwent coronary angiography and CMR were analyzed. All patients provided written informed consent for participation in the REVEAL Trial. The Institutional Review Board of Duke University Medical Center approved the present sub-study.

### Angiographic analysis

An independent interventional cardiologist, blinded to clinical data and CMR results, reviewed coronary angiograms from the REVEAL trial. The IRA was determined based on angiographic parameters including visually significant stenosis (≥70%), presence of thrombus and TIMI flow.[7] The IRA was classified as the left anterior descending artery (LAD), the right coronary artery (RCA) or the left circumflex artery (LCx). The REVEAL trial included only patients with an IRA of a major epicardial coronary artery or its large branches, with TIMI grade 0 or 1 flow, and was classified according to the parent vessel. After restoration of TIMI 2 or 3 flow, the reader identified the myocardial segments supplied by the IRA and coded them according to the standardized 17-segment segmentation model.[8] The LVEF was visually estimated by left ventriculography when performed. MVD was defined as ≥70% stenosis in greater than one major epicardial vessel.

### CMR acquisition, processing, and analysis

CMR scans were performed at 2–6 days post presentation. Patients were placed supine in a 1.5-T clinical scanner (Siemens, Sonata, Erlangen, Germany), and a phased-array receiver coil was placed on the chest for imaging. Cine images were acquired in six to ten short-axis views and two long-axis views. Short-axis views were obtained just below the level of the mitral-valve insertion plane and then every 1 cm throughout the left ventricle. A gadolinium-based contrast agent was then administered intravenously, and contrast-enhanced images were acquired in the same views as those for cine-CMR. DE images were acquired with a segmented inversion-recovery gradient-echo pulse sequence.[8, 9] The typical inversion delay was 300–350 ms and the typical voxel size was 1.9 x 1.4 x 6 mm. Quantitative T2-mapping was performed in all subjects using a T2-prepared steady-state free precession sequence[10] with the following imaging parameters: TR/TE 240/1.19 ms, flip angle 70 degrees, field of view 270×185 mm^2^, matrix 192×132 pixels, slice thickness 6 mm, parallel imaging factor 2, acquisition in late diastole on every fourth heartbeat, T2 preparations; 0 ms, 24 ms, 55 ms, 90 ms, size 1.9×1.4×6.0 mm, temporal resolution 239 ms.

The CMR scans were analyzed by one blinded observer blinded to clinical data and CA results using the identical 17-segment model as for CA.[11] Cine-CMR was used for the visual assessment of LVEF and the presence or absence of an aneurysm. Delayed enhancement (DE)-CMR was used for the assessment of MI (both left and right ventricle) by presence-absence on visual assessment. In addition, the presence of microvascular obstruction (MVO) and left ventricular thrombus was noted. Although T2 weighted imaging was performed in all subjects, the presence of T2-weighted edema and/or MVO was used in patients with multiple infarcts to determine acuity of infarction by visual analysis. Multiple infarcts in the same patient were defined as the presence of hyperenhanced tissue in two distinct coronary artery territories.

### Comparison of angiographic and CMR data

For the determination of a match between the IRA by CA and the infarct territory on CMR, the segments affected by the IRA as coded according to the 17-segment model on CA were compared to the segments of hyperenhancement noted on DE-CMR. A match was defined as overlap of at least one segment of any of the 17-segments. To account for possible misregistration two independent research investigators re-analyzed both the CA and CMR side by side to confirm a mismatch.

### Statistical analysis

As this manuscript is purely descriptive in nature, no formal statistical analysis was performed.

## Results

The present analysis included 113 patients with a median age of 57.3 years and 21 (19%) women. A total of 17 (15%) had a history of diabetes, 54 (48%) had hypertension and hyperlipidemia. Analysis of angiographic images revealed that in patients presenting with STEMI, 63% experienced an acute infarct to the RCA, 27% to the LAD, and 10% to the LCx. The average ejection fraction measured on CMR was 47% (±11) and 43% (±14) by angiography. (Table 1)

**Table 1:** Baseline Characteristics of Patients.

	Overall (n = 113)
**Demographics**	
Age (years), Median (25^th^, 75^th^)	57.3 (49.3,66.4)
Female sex,	21 (18.6%)
*Ethnicity*	
Hispanic or Latino	4 (3.5%)
Not Hispanic or Latino	109 (96.5%)
*Race*	
American Indian or Alaska Native	0
Asian	3 (2.7%)
Black or African American	15 (13.3%)
Native Hawaiian or other Pacific Islander	1 (0.9%)
White	93 (82.3%)
Other	1 (0.9%)
**Medical History**	
Hypertension	54 (47.8%)
Peripheral Vascular Disease	3 (2.7%)
Diabetes	17 (15.0%)
Hyperlipidemia*	54 (47.8%)
Family history of premature CAD^†^	34 (31.8%)
Severe chronic obstructive pulmonary disease	3 (2.7%)
Chronic Kidney Disease^‡^	0
Hepatic impairment^§^	0
Cancer within the last 5 years^| |^	4 (3.5%)
**Cigarette smoking**	
Never	44 (39.6%)
Current smoker,	38 (34.2%)
Quit < 6 months ago,	6 (5.4%)
Quit ≥ 6 months ago,	23 (20.7%)
**Cardiac medications Used at Baseline,**	
Aspirin	101 (89.4%)
Clopidogrel	107 (95.5%)
Unfractionated heparin	96 (85.7%)
Low molecular weight heparin	8 (7.1%)
GP IIb/IIIa inhibitors	78 (69.0%)
Thrombolytic therapy	10 (8.8%)
Oral anticoagulant	3 (2.7%)
**Vital Sign/Physical Assessment**	
Height (cm) Median (25^th^, 75^th^)	175.0 (168.0,180.0)
Weight (cm) Median (25^th^, 75^th^)	87.6 (73.6,97.3)
Systolic Blood Pressure (mmHg) Median (25^th^, 75^th^)	126.0 (116.0,141.0)
Diastolic Blood Pressure (mmHg) Median (25^th^, 75^th^)	79.0 (71.0,87.0)
Pulse (beats/min) Median (25^th^, 75^th^)	75.0 (66.0,86.0)
*Killip Class*	
I	102 (98.1%)
II	2 (1.9%)
*TIMI Grade at Start of Angiography N*	
0	99 (87.6%)
1	14 (12.4%)
**Procedural & Timing Information**	
*Type of PCI*	
Primary	99 (87.6%)
Rescue	14 (12.4%)
*Infarct Related Artery N*	
Non-Anterior	82 (72.6%)
Anterior	31 (27.4%)
*Culprit Vessel*	
Right Coronary Artery	71 (62.8%)
Left Main	0
Left Anterior Descending	31 (27.4%)
Left Circumflex	11 (9.7%)
*TIMI Grade After PCI*	
2	2 (1.8%)
3	111 (98.2%)
*Were Stents Used*?	
Yes	111 (98.2%)
No	2 (1.8%)
*Were Drug Coated Stents Used*?	
Yes	45 (40.9%)
No	65 (59.1%)
*Was a Thrombectomy Device Used*?	
Yes	38 (33.6%)
No	75 (66.4%)
Time from symptom onset to TIMI flow restoration (min) Median (25th, 75th)	180.0 (121.0,250.0)
Time from symptom onset to study drug admin. (min) Median (25th, 75th)	375.0 (296.0,450.0)
Time from TIMI flow restoration to study drug admin. (min) Median (25th, 75th)	178.0 (124.0,225.0)
Time PCI stopped to study drug admin. (min) Median (25th, 75th)	153.0 (90.0,205.0)
Time from hospitalization to TIMI flow restoration (min) Median (25th, 75th)	67.0 (41.0,109.0)
Time from hospitalization to randomization (min) Median (25th, 75th)	211.5 (151.0,268.5)
Time from randomization to study drug admin. (min) Median (25th, 75th)	30.0 (18.0,48.0)
Time from TIMI flow restoration to randomization (min) Median (25th, 75th)	136.5 (81.0,185.0)

Abbreviations: CAD, coronary artery disease;

* cholesterol > 200 mg/dL, or LDL > 100 mg/dL or requiring medication;

^†^CAD before age 55;

^‡^ known disease and/or creatinine > 177 mmol/L or 2.0 mg/dL;

^§^ known impairment and/or ALT > 3 X upper limits of normal;

^| |^ excluding skin cancer

### Correlation between IRA on angiography and CMR

Analysis of the agreement between the IRA identified by CA and the infarct territories by CMR found a total of 5 (4%) patients to have a mismatch. In four of the five cases, there were multiple infarcts noted on CMR. With the use of T2-weighted edema imaging and microvascular obstruction on DE-CMR, the acuity of each infarct could be determined. Interestingly, the IRA identified by CA in these four cases were in an area of chronic infarct. In the fifth case, the coronary artery supplying the infarcted myocardium by CMR did not show any significant stenosis, and the IRA selected by CA was in a different territory that had no infarct on CMR.

**Fig 1:**
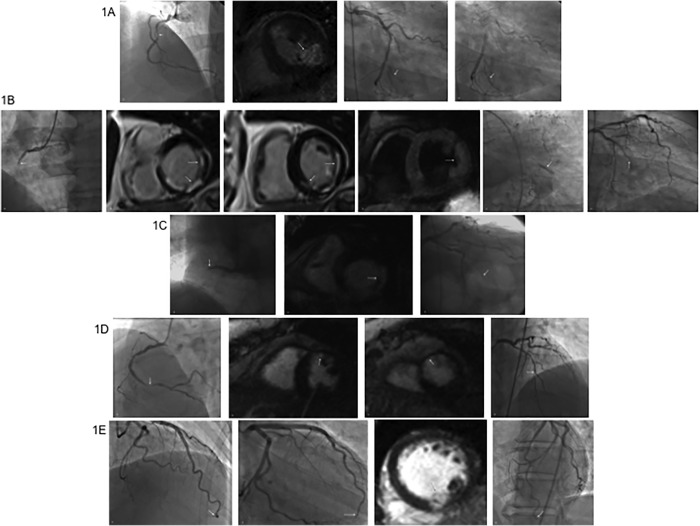
Mismatch between Cardiac Magnetic Resonance and Angiography. **1A:** The clinical interpretation of the IRA was the RCA (white arrow), (A) which underwent PCI with stent placement. The IRA was indeterminate by blinded analysis. CMR showed an inferolateral wall infarct (white arrow) (LCx distribution) (B). With the CMR data available, reviewing the angiography data again, the OM was seen to have a flush occlusion that was filled retrograde (white arrows) (C and D). **1B:** The clinical and blinded interpretation of the IRA was the RCA, which underwent PCI with stent placement (white arrow) (A). CMR showed infarcts in both the RCA and LCx distribution (white arrows) (B and C). T2 weighted imaging indicated that the OM territory was acute (white arrow) (D). With the CMR data available, angiography revealed retrograde filling of OM (white arrows) (E and F). **1C:** The clinical and blinded interpretation of the IRA was the RCA, which underwent PCI with stent placement (white arrow) (A). CMR showed an infarct in the inferolateral (LCx) distribution with no scar in the inferoseptal wall (white arrow) (B). There was also retrograde filling of the OM (white arrow) (C). **1D:** The clinical and blinded interpretation of the IRA was the RCA, which underwent PCI with stent placement (white arrow) (A). CMR showed an infarct in the mid-distal anterior wall (LAD distribution) (white arrows) (B and C). On cardiac angiography, there was stenosis noted in the mid-LAD territory (white arrow) (D). **1E:** The clinical and blinded interpretation of the IRA was the LAD, which underwent balloon angioplasty (white arrow) (A and B). CMR showed an infarct in the inferoseptal wall, (gray arrow) (C) which does not correlate with the area of distribution of the distal LAD. This area of distribution, is attributed to the PDA, which originates from the LCx, however no obvious stenosis was appreciated in that region (white arrow) (D).

In the first identified mismatch, the IRA was determined to be the RCA, which underwent PCI with stent placement. On initial blinded analysis, the IRA was indeterminate. CMR showed an infarct in the inferolateral wall. It was only in hindsight, after reviewing the CMR and looking again at angiography, that a flush occlusion with retrograde filling was identified in the obtuse marginal (OM) branch of the LCx (Fig 1A). In the second and third cases, the clinical and blinded interpretation of the IRA was the RCA, which underwent stent placement. On CMR, there were infarcts identified in both the RCA and LCx territories. Using T2-weighted imaging in the second case and MVO in the third case, the OM territory was identified as acute. Review of angiography with CMR data revealed retrograde filling of the OM in both cases (Fig 1B and 1C). In the fourth case, the RCA was identified as the IRA by clinical and blinded analysis and underwent stent placement. CMR indicated an infarct in the mid-distal anterior wall, correlating to the LAD and review of angiography indicated stenosis in the mid-LAD territory (Fig 1D). The IRA in the fifth mismatch case was identified as the LAD by CA which underwent balloon angioplasty however CMR revealed an infarct in the inferoseptal wall, attributed to the PDA which originated from the LCx, however, there was no obvious stenosis appreciated in that region (Fig 1E).

### Multiple infarcts

**Fig 2:**
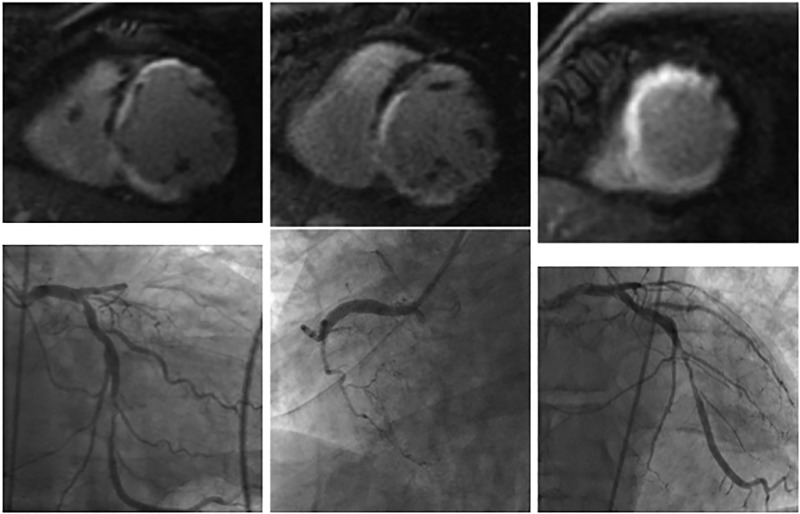
Multiple Infarcts on CMR and CA. CMR with evidence of multiple infarcts in LAD, RCA, and LCx distribution (A-C). Angiography with high-grade stenosis in LCx and LAD, 100% stenosis RCA (D-F)

In this trial cohort of patients without prior known MI (n = 113), 13 patients (11.5%) had multiple infarcts in separate territories on CMR with 4 patients (3.5%) having multiple acute infarcts and 9 patients (8%) having both acute and chronic infarcts. Of the multiple acute and chronic infarcts 3 involved all three vessels (RCA, LAD, LCx) and 6 involved the RCA and LCx. (Fig 2)

### RV infarcts

**Fig 3:**
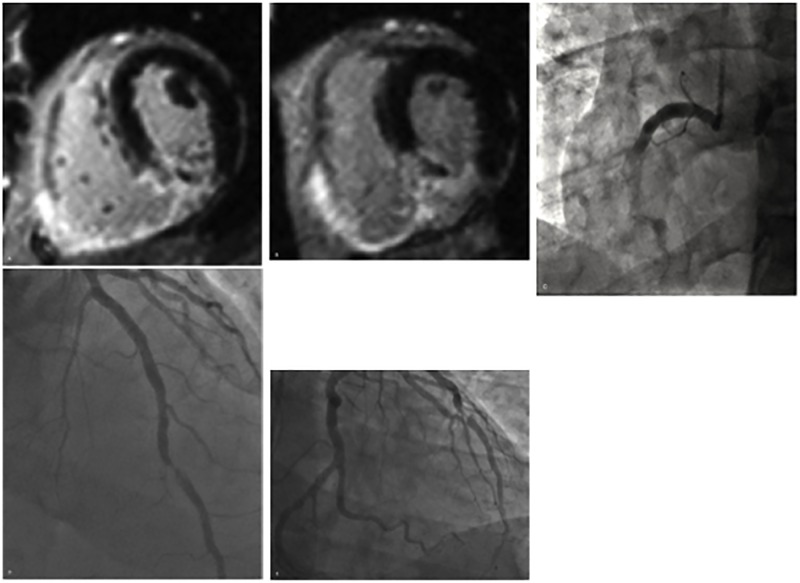
Right Ventricular Infarct on CMR and CA. CMR with evidence of RV infarct (A and B). Angiography demonstrating RCA stenosis (C-E).

Twenty-three patients (20%) with STEMI exhibited right ventricular infarction. Among the 62 patients with the IRA as the RCA, 21 (34%) experienced right ventricular infarcts (RVI). Additionally, there were two patients who had an infarct in the right ventricle from an IRA that was identified from the LAD and one from the LCx, however, both were left dominant coronary anatomy. (Fig 3)

### Thrombus, microvascular obstruction, aneurysms

Among patients in the present study, 9 (8%) were found to have aneurysms and 10 (9%) were found to have the presence of left ventricular thrombi. There were a total of 44 (39%) patients with microvascular obstruction (MVO) on CMR images of which, 22 (50%) were RCA infarcts, 17 (39%) were LAD infarcts, and 13 (30%) were LCx infarcts. In the analysis of coronary dominance, 78% of patients were found to be right dominant, 12% were left dominant, and 10% were co-dominant.

## Discussion

The present study examined patients presenting with STEMI who had TIMI flow grade 0 or 1 and no prior history of PCI to the IRA and no prior MI. We found a mismatch between the IRA identified by CA and CMR in five patients. Additionally, we found a relatively high incidence of RVI and multiple infarcts. The lack of ECG data in the present analysis is a potential major limitation, however, in all five mismatch cases, the clinical team who had access to the ECG intervened on an artery that was not identified as the IRA as defined by CMR.

In one case, we could not identify the IRA on CA even with the information provided by CMR. A few potential explanations for this finding are: a) vasospasm, b) spontaneous thrombolysis prior to angiography being performed, or c) flush occlusion of a branching vessel, which could not be visualized by angiography. This patient did not have multiple territories of infarction by CMR and thus misinterpretation of the acute lesion could not explain this finding.

An interesting observation from this study is that MVD with at least one vessel being completely occluded was seen in 4 of the 5 cases and could be a potential avenue for when the IRA is missed. It has been shown that up to half of patients with STEMI present with MVD, associated with increased risk of morbidity and mortality.[12–14] Our study demonstrated an 11.5% incidence of MVD, lower than previous reports. However, this is likely related to the strict inclusion criteria. This suggests that there is perhaps an even higher incidence of mismatch in the broader population of patients presenting with STEMI (ie. patients with previous revascularization or presenting with TIMI grade 2 or 3 flow). Recent studies have demonstrated improved cardiovascular outcomes with preventive PCI,[15–17] contributing to the allowance for consideration of MVD PCI, either as a planned, staged procedure or at the time of primary PCI according to the recently updated guidelines.[3] The results of this study raises the question that perhaps part of the benefit of performing complete revascularization in patients presenting with STEMI, may be due to the higher likelihood of revascularizing the correct IRA. Although this study found only 4% mismatch, it is likely that there would be a larger percentage of mismatch in the broader population without the strict entry criteria of REVEAL. Perhaps a strategy that might be worth testing would be that in patients presenting with STEMI and MVD, after intervening emergently on the IRA as determined by the interventionalist, CMR is then performed to assess the area of infarction to confirm the correct IRA was chosen and if not, further revascularization would be performed. However this would need to be further validated in large studies prior to incorporation into clinical practice.

In reviewing the CMR images, we determined that 4 patients had more than one acute territory of infarct as determined by T2-weighted imaging and/or MVO. Plaque instability associated with acute myocardial infarction is not a local event but may in fact be associated with a diffuse process, occurring throughout the coronary anatomy.[18, 19] Among patients with acute MI, there is diffuse activation of inflammatory cells not only in the IRA but in non-IRA vessels as well.[20] Angioscopic imaging of patients with acute MI reveal that vulnerable plaques are present in both culprit and non-culprit coronary arteries.[21] The presence of multiple complex coronary plaques is associated with worse outcomes that include recurrence of angina, MI, and greater reduction in left ventricular function.[18]

Right ventricular infarction in acute myocardial infarction is a poor prognostic indicator, associated with cardiogenic shock, ventricular arrhythmias, and advanced atrioventricular block.[22] Contrast enhanced CMR imaging allows for superior identification of right ventricular infarcts.[23–27] Right ventricular injury is not limited to inferior infarcts, but can be found in anterior infarcts as well.[23] A recent study showed that the right ventricle was involved in 46% of patients with inferior infarcts and 30% of anterior infarcts.[24] In the present study, although RVI was primarily present in RCA infarcts, it was found in the LAD and LCx, however the incidence was much lower in our study likely due to the stringent inclusion criteria.

### Limitations

The present study looked at a very specific subset of patients, as noted by the fewer anterior infarcts compared to the general population, with TIMI flow grade 0 or 1 and no prior history of MI or revascularization to the IRA. Therefore, it is likely that with a broader STEMI population, there may be an even higher incidence of mismatch. Another limitation to this study is the challenges of mapping the myocardial territory supplied by the IRA on a 17-segment model and the potential for misregistration. To limit this potential methodological challenge, we only classified a mismatch after meticulously reviewing each mismatch case with angiography and CMR images side by side. An additional major limitation is the qualitative as opposed to quantitative analysis of CMR images. An important consideration is that in patients with a chronic occlusion of a vessel within the identified infarct region, supplied from collaterals from a neighboring territory, a sudden occlusion of the neighboring, collateral-supplying vessel, may in fact be the culprit, infarct related artery, and an appropriate intervention was in fact performed clinically. As mentioned previously, a potential major limitation to this study is that the blinded interventional cardiologist did not have access to the ECG, however, the clinical team who did have access to the ECG, intervened on the vessel that was not determined by CMR to be the IRA in all five of the cases, thus suggesting that this did not impact the results of our study.

## Conclusions

In this select population of patients who met the strict entry criteria of the REVEAL study, the identification of the IRA by CA was incorrect in 4% of patients presenting with STEMI. Four patients with a mismatch had an acute infarction in more than one coronary artery territory on CMR. The role of CMR in patients presenting with STEMI with MVD on CA deserves further investigation.

## Supporting Information

S1 Appendix Study Data Set. (XLSX)
